# Preliminary Comparison of the Efficacy and Safety of Needle-Embedding Therapy with Acupuncture for Atopic Dermatitis Patients

**DOI:** 10.1155/2019/6937942

**Published:** 2019-04-23

**Authors:** Ho-Chan Lee, Soo-Yeon Park

**Affiliations:** Department of Ophthalmology, Otolaryngology & Dermatology, College of Korean Medicine, Dongshin University, 185 Geonjae-ro, Naju-si, Jeollanam-do, Republic of Korea

## Abstract

**Objectives:**

Among Traditional Korean Medicine approaches, needle-embedding therapy is used in various fields and consistently studied; however, there have been no clinical studies of the treatment of adult atopic dermatitis (AD) with needle-embedding therapy. Thus, there is a need to investigate the effects of needle-embedding therapy for treatment of AD. This study was performed to identify possible effects of needle-embedding therapy at Quchi acupoint (LI11) on AD and to compare these effects with those of acupuncture therapy.

**Methods:**

A total of 14 participants were enrolled in this study. Participants received acupuncture or needle-embedding treatments for 4 weeks and then were followed for an additional 2 weeks because of safety assessment. The participants were divided into 2 groups: the acupuncture group, receiving treatment at Quchi acupoint (LI11) 3 times per week, and the needle-embedding group, receiving treatment at Quchi acupoint (LI11) once per week. The groups were compared on the basis of the SCORing Atopic Dermatitis (SCORAD) index, Transepidermal Water Loss (TEWL), skin hydration, and Dermatology Life Quality Index (DLQI) at baseline and 1 week after treatment was completed (5th week).

**Results:**

The SCORAD index, TEWL, Skin hydration, and DLQI at 1 week after treatment were significantly improved in both groups (p<0.05). However, there were no significant differences between the acupuncture and needle-embedding groups in any of the main evaluation indices (p>0.05). The study participants received a total of 84 acupuncture treatments or 28 needle-embedding treatments. No adverse events occurred during the study period.

**Conclusions:**

Based on changes in the SCORAD index, TEWL, skin hydration, and DLQI value, we found that both needle-embedding and acupuncture treatments at the Quchi acupoint (LI11) were effective in decreasing the symptoms of AD and exhibited similar therapeutic effects, which suggests that needle-embedding treatment may be more clinically convenient than acupuncture, with longer effects and fewer treatments.

## 1. Introduction

Atopic dermatitis (AD) is a chronic, recurrent eczema that is accompanied by itching and typically begins in infants. The prevalence and appearance of disease lesions, according to age, has been increasing worldwide [1, 2]. The primary cause of AD is not yet clear; however, it is suspected to have a multifactorial pathogenesis, including genetic defects [3–5]. Recently, the cause of AD was reported as abnormal immune response activation after damage to the skin barrier, caused by genetic mechanisms [6].

There are many clinical aspects of AD. In Korea, Hanifin and Rajka's original diagnostic criteria [7] were modified based on clinical patterns of Koreans by the Korean Academy of Skin Sciences in 2005 [8]; these modified criteria are widely used in Korea. For treatment of AD, Western medicine has typically used a combination of emollients, corticosteroids, antibiotics, calcineurin inhibitors, UV phototherapy, and systemic immunomodulating therapies, such as cyclosporine and interferon gamma-1b [2, 9, 10]. However, this cannot be the fundamental treatment for AD; interest in Traditional Korean Medicine treatment has increased because of various side effects associated with long-term use of steroids, such as skin atrophy or potential growth delay [11].

In Korea, patients with AD have frequently used Traditional Korean Medicine, such as acupuncture and herbal medicine, for symptom management [12]. Regarding the use of acupuncture for AD, studies of the effects of acupuncture treatments have been published overseas [13–17], as well as in Korea [18–21]. Among Traditional Korean Medicine approaches, needle-embedding therapy is used in various fields and consistently studied; however, there have been no clinical studies of the treatment of AD with needle-embedding therapy. Thus, there is a need to investigate the effects of needle-embedding therapy for treatment of AD. We conducted a preliminary comparative study, in preparation for future clinical trials. This study was performed to identify possible effects of needle-embedding therapy at Quchi acupoint (LI11) on AD and to compare these effects with those of acupuncture therapy.

## 2. Materials and Methods

This was a preliminary comparative study for future clinical trials. Based on the results of this study, we will design further clinical studies and obtain approval for additional complementary research. The study design was a single-center, single-blinded (observer), prospective, randomized clinical pilot trial with an additional experimental portion. The acupuncturist and observer were different members of the clinical research team.

### 2.1. Participants and Recruitment

This clinical study was performed from January 01, 2018, to July 31, 2018, at the Department of Dermatology of the Oriental Hospital of Dongshin University. Participants were recruited by a clinical research recruitment announcement. Participants provided written consent after they were informed of the purpose and contents of the clinical study. This study was approved by the IRB of the Korean Oriental Medical Center (IRB Approval No. 2017-12); the specific inclusion and exclusion criteria are as follows.

(1) Participants who met all of the following criteria were included:

1. Those with age between 20 and 50 years
2. Those who met the Hanifin and Rajka criteria, with consistent AD symptoms for at least 3 months prior to study enrollment
3. Those with SCORing Atopic Dermatitis (SCORAD) index score 10–40
4. Those not taking prescription drugs for AD for at least 1 month prior to enrollment
5. Those able to understand the study protocol and voluntarily agree to participate
6. Those not participating in any other research studies for AD for at least 1 month prior to enrollment

(2) Participants who met any of the following criteria were excluded:

1. Those with a fear of acupuncture and needle-embedding therapy
2. Those using treatments that may affect study outcomes (e.g., oral corticosteroids)
3. Those with asthma or bronchitis
4. Those with other disorders that may affect study outcomes (e.g., anxiety or depression)
5. Those with a heart disease (e.g., heart failure, angina pectoris, or myocardial infarction)
6. Those who were pregnant, lactating, or not using sufficient contraception (women only)
7. Those who were unfit to participate in the study, as determined by the principal investigator

### 2.2. Randomization and Allocation Concealment

After providing written consent, participants underwent a screening test to confirm eligibility. The baseline characteristics including demographics, physical examination, medical history, and severity of AD signs were used to screen eligible participants according to the inclusion and exclusion criteria. Individuals with AD were randomly assigned to 1 of 2 groups via block randomization.

### 2.3. Blinding and Code Breaking

Participants and the outcome assessor were blinded to the treatment allocation in this trial. Because it was impossible to blind the Korean Medicine Doctors (KMDs) who provided acupuncture and needle-embedding treatments, the KMDs did not perform outcome measurements or analyze data.

### 2.4. Interventions

A total of 14 participants were enrolled in this study. Participants received acupuncture treatment or needle-embedding treatment for 4 weeks. The participants were divided into 2 groups: an acupuncture group that received treatment 3 times per week and a needle-embedding group that received treatment once per week.

Acupuncture and needle-embedding treatments were performed by KMDs who had more than 2 years of clinical experience after graduating from Korean medical school. The insertion area was wiped once or twice with alcohol-soaked cotton before and after the procedure. Previously administered medication was permitted during the test period; no other additional treatments were performed. However, for patients who required concomitant use of drugs, qualification for participation was validated. When necessary, an assessment was conducted by the person in charge of testing, in accordance with the following criteria.

#### 2.4.1. Acupuncture Treatment

The acupuncture protocols used in this study reflected everyday clinical practice in Korean Medicine; manual acupuncture treatment was performed on the basis of the traditional meridian theory and consensus by experts in acupuncture and AD.

The acupuncture group received a total of 12 acupuncture treatments three times per week for 4 weeks. Acupuncture needles (0.25 × 40 mm, stainless steel needle, Dongbang Co., Korea) were used for treatment; acupuncture treatment was performed at the bilateral Quchi acupoint (LI11) and retained for 20 minutes. The bleeding area was then compressed and sterilized. Manipulation was not used.

#### 2.4.2. Needle-Embedding Treatment

The needle-embedding group received a total of 4 needle-embedding treatments, once per week for 4 weeks. We used sterilized needle-embedding acupuncture needles (31G × 25 mm), Miracu Needle-embedding acupuncture (DB Medical, PALTEC CORPORATION). Needle-embedding acupuncture was performed at the bilateral Quchi acupoint (LI11). The procedure was performed in the order of incision, stinger, and sedimentation; the bleeding area was then compressed and sterilized.

### 2.5. Outcome Assessments

The SCORAD index, Transepidermal Water Loss (TEWL), Skin hydration, and Dermatology Life Quality Index (DLQI) were used to measure outcomes. The SCORAD index was used as the primary outcome measure.

#### 2.5.1. SCORAD Index

The SCORAD index is a widely used tool to assess the severity of AD. The SCORAD index evaluates the intensity and extent of affected regions; it also estimates subjective discomfort, such as pruritus and sleep loss symptoms [22]. The SCORAD index contains 6 items to evaluate AD intensity: erythema, excoriation, edema or papulation, lichenification, oozing or crust, and dryness. In this index, the extent of the lesions is evaluated as a percentage of the entire external surface of the human body, using the rule of nine. The SCORAD index evaluates the intensity and extent of affected regions and then estimates subjective discomfort, such as pruritus and sleep loss symptoms. The SCORAD index has been used in many studies of AD in Korea; it was developed by the European Task Force on Topic Dermatitis in 1993. It has been the most popular method of evaluation and is recommended as an appropriate method of assessment for AD [23]. The SCORAD index classifies less than 15 points as mild, less than 40 points as moderate, and more than 40 points as severe [24].

#### 2.5.2. Transepidermal Water Loss (TEWL)

The Tewameter TM300 (Courage+Khazaka electronic GmbH, Germany) was used to measure TEWL at 1 cm  below the Quchi acupoint (LI11). The TEWL is most often used as an index for evaluating the permeability barrier function of the stratum corneum by calculating the water slope formed on the surface of the skin by the water evaporated through the epidermis. It measures the epidermal permeability barrier function of the skin by measuring the passive diffusion of water vapor on the surface of the skin. By Fick's law, the water vapor is assumed to diffuse from the skin surface to the outside; the vapor pressure can be calculated, as measured through 2 sensors at a certain distance from the chamber in the probe closely attached to the skin surface. Thus, because the measured value is the speed of evaporation of water, the unit is g/h/m^2^; a lower resulting value is considered an indicator of healthier permeation barrier function [25].

The isothermal-isohumidity conditions were set at 20-25°C indoor temperature and 40-60% indoor humidity. The patients were stabilized in the clinical laboratory for approximately 30 minutes prior to measurement. For the convenience of measurement, TEWL was measured at 1 cm  below the Quchi acupoint. The probe was placed on the measurement area and measurements were continuously recorded at 1-second intervals until the displayed value was stabilized; the final 5 measurements were averaged and evaluated.

#### 2.5.3. Skin Hydration

Skin-O-Mat (CM825, CK electronics, Germany) was used to measure skin hydration at the lower 1 cm  of the Quchi acupoint (LI11). For the convenience of measurement, skin hydration was measured at 1 cm  below the Quchi acupoint. The measurement of skin hydration was performed after measurement of TEWL, and was analyzed using the program MPA5 (CK Electronics, Germany). In order to minimize measurement error, the average skin hydration was recorded in 2 consecutive measurements. The unit of measurement is expressed as an Arbitrary unit (AU) given by the device; a higher reading indicates higher skin surface moisture content [26]. A KMD performed measurements to eliminate bias among the measurers.

#### 2.5.4. Dermatology Life Quality Index (DLQI)

The DLQI was developed in 1992 by Finlay and Kahn for use as a clinical tool to assess the impact of skin diseases on patients' quality of life [27]. Lee et al. [28] translated the DLQI into Korean and showed that DLQI can be an important parameter for evaluating the disease course in AD patients. In this study, the Korean translation of Finlay and Khan was used with the consent of the authors.

The DLQI comprises 10 questions, with categories related to the effects of treatment on symptoms and feelings, daily activities, leisure, work and school, personal relationships, and treatment. Each question is rated from 0 to 3, and the total score is 0 to 30; a higher score indicates worse quality of life. Specific scores are as follows: 0-1 is no effect, 2-5 is small effect, 6-10 is moderate effect, 11-20 is large effect, and 21-30 is extremely large effect.

### 2.6. Safety Assessment

An investigation of the following adverse reaction was conducted after each treatment and 1 week after the treatment was completed. Two weeks after the treatment were completed, an adverse reaction survey was conducted by phone.

- External body reactions (e.g., erythema, pruritus, fever, edema, subcutaneous bleeding, discoloration, hardening, and pus formation)
- Subjective symptoms (e.g., pain, nausea and dizziness, cold sweat, hyperventilation, heart palpitation, sensory impairment, and headaches)
- Skin system (e.g., local paralysis, local edema, and blisters)
- Musculoskeletal pain and other reactions

### 2.7. Statistical Analyses

Baseline characteristics were compared between the 2 groups. Continuous data are presented as means and standard deviations; these were compared using the independent t-test or Wilcoxon's rank-sum test. Categorical data are presented as frequencies and percentages; these were compared using the chi-squared test or Fisher's exact test.

Dependent variables included values measured before intervention and 1 week after the completion of intervention. A Mann-Whitney* U* test was conducted to detect differences between therapies. A p value of < 0.05 was considered significant; participants who dropped out of the study were excluded from the analysis (i.e., a per-protocol analysis was performed). All statistical analyses were performed using SPSS version 22.0.

## 3. Results

### 3.1. Study Participants

**Figure 1:**
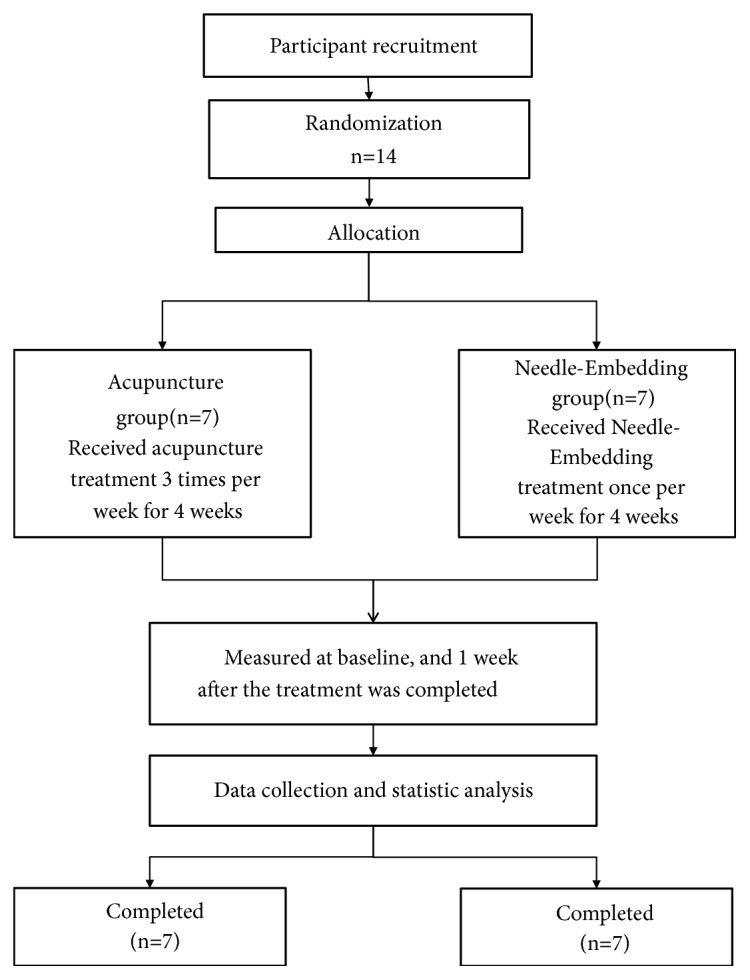
Flow chart of the trial.

A total of 14 participants were enrolled in this study. Of these 14 participants, none dropped out (Figure 1).

### 3.2. Baseline Characteristics

The baseline characteristics did not show significant imbalances between the 2 groups; there were similar risk factors for AD, such as age, drinking, and smoking. There were no significant differences between the 2 groups in the ratio of men to women (acupuncture treatment group: 1 man (14.3%) and 6 women (85.7%), needle-embedding therapy group: 1 man (14.3%) and 6 women (85.7%)), in age (acupuncture group: 34 ± 5.68 years old, needle-embedding therapy group: 30.71 ± 7.57 years old; mean ± SD), in smoking status (acupuncture group: Yes (0, 0%), No (7, 100%), needle-embedding therapy group: Yes (1, 14.3%), No (6, 85.7%)), or in drinking status (acupuncture group: Yes (4, 57.1%), No (3, 42.9%), needle-embedding therapy group: Yes (3, 42.9%), No (4, 57.1%)) (Table 1).

### 3.3. Outcome Variables

#### 3.3.1. SCORAD Index

The SCORAD index at baseline showed no significant difference between the 2 groups (p=0.405). The SCORAD index at 1 week after treatment was significantly improved in both groups (p<0.05) (needle-embedding treatment group: from 26.1 ± 5.24 to 17.0 ± 4.16; acupuncture treatment group: from 23.9 ± 3.89 to 15.1 ± 3.18; mean ± SD). However, there was no significant difference between the acupuncture and needle-embedding treatment groups (p=0.697) (Table 2).

#### 3.3.2. TEWL

The TEWL at baseline showed no significant difference between the 2 groups (p=0.157). The TEWL at 1 week after treatment was significantly improved in both groups (p<0.05) (needle-embedding treatment group: from 9.1 ± 1.52 to 5.4 ± 1.04 g/h/m^2^; acupuncture treatment group: from 11.7 ± 4.96 to 5.9 ± 1.22 g/h/m^2^; mean ± SD). However, there was no significant difference between acupuncture and needle-embedding treatment groups (p=0.306) (Table 3).

#### 3.3.3. Skin Hydration

Skin hydration at baseline showed no significant difference between the 2 groups (p=0.749). Skin hydration at 1 week after treatment was significantly improved in both groups (p<0.05) (needle-embedding treatment group: from 24.7 ± 3.84 to 34.4 ± 6.40 AU; acupuncture treatment group: from 24.5 ± 2.40 to 33.7 ± 7.06 AU; mean ± SD). However, there was no significant difference between acupuncture and needle-embedding treatment groups (p=0.949) (Table 4).

#### 3.3.4. DLQI

The DLQI at baseline showed no significant difference between the 2 groups (p=0.476). The DLQI at 1 week after treatment was significantly improved in both groups (p<0.05) (needle-embedding treatment group: from 5.6 ± 1.90 to 1.0 ± 1.73; acupuncture treatment group: from 5.3 ± 3.40 to 1.1 ± 0.69; mean ± SD). However, there was no significant difference between acupuncture and needle-embedding treatment groups (p=1.000) (Table 5).

### 3.4. Safety

The study participants received a total of 84 acupuncture treatments or 28 needle-embedding treatments. No severe adverse events occurred during the study period.

## 4. Discussion

Needle-embedding acupuncture therapy is a newly designed acupuncture technique that uses a specially designed device to embed certain substances in the body, thus infusing a sustained stimulation effect [29]. Needle-embedding therapy can be applied for all diseases, such as acute or chronic diseases, as well as Excess or Deficiency Syndrome, by freely adjusting the stimulation time and intensity at the acupoint [30]. In Korea, there have been studies regarding facial nerve palsy [31], the effects of facial needle-embedded acupuncture therapy on skin elasticity [32], facial wrinkle improvement [33], and comparison of the effects of acupuncture and needle-embedded acupuncture in headache patients [34]. However, there have been no clinical studies of AD treatment with needle-embedding acupuncture therapy.

Acupuncture therapy has been increasingly used in treatment of AD worldwide, and several clinical studies have demonstrated the therapeutic effects of acupuncture [13–20]. Although these studies seemed to indicate that acupuncture has therapeutic effects on major characteristics of AD, no RCTs have been performed to evaluate the effects of acupuncture on AD as a specific disease entity. Moreover, there has been a lack of placebo-controlled studies regarding sham acupuncture. However, a recent study with sham acupuncture served as a placebo-controlled trial; it showed that acupuncture improved symptoms in patients with AD [21].

The treatment acupoint for AD is the Quchi acupoint (LI11), which is the typical acupoint used in skin diseases [35, 36]. In addition, the Atopic Dermatitis Korean Medicine Clinical Practice Guideline recommends the Quchi acupoint (LI11) at the GPP level [37]. This study was performed to characterize the effects of needle-embedding therapy at the Quchi acupoint (LI11) on AD and to compare these effects with those of acupuncture therapy.

In order to confirm the efficacy and safety of needle-embedding acupuncture therapy and acupuncture, we have previously conducted clinical studies [38, 39]. Our results showed that acupuncture is effective in moisturizing skin by improving TEWL; furthermore, needle-embedding acupuncture therapy has been reported as safe when used for treatment of skin dryness.

The SCORAD index, used in many studies of AD in Korea [23], was selected as the primary evaluation criterion. TEWL and Stratum Corneum Hydration are the most commonly used indicators for evaluating skin barrier function. In addition to the advantages of noninvasive methods, a number of prior studies reporting a high correlation between skin barrier function and severity of skin diseases suggest that measurement of skin moisture can be used as a tool for diagnosing skin diseases [40, 41].

AD, along with psoriasis, affects the patients' quality of life more than any other skin disease. It causes discomfort and mental pain in everyday life, interferes with normal interpersonal relationship and social activities, and affects the quality of life of patients' immediate family members [42]. Currently, the medical community has emphasized that the severity of the disease should be measured and evaluated and that the patient's quality of life should be assessed [43].

Taken together, the SCORAD index, TEWL, skin hydration, and DLQI were statistically significantly different between baseline and the 5-week follow-up assessment (p<0.05). These results suggest that both needle-embedding and acupuncture treatments can improve the symptoms of AD patients and may also improve their skin barrier function and quality of life. These results may be caused by the antiallergic [13, 14], anti-inflammatory [18], and antipruritic effects [44] of acupuncture. Needle-embedding therapy is also suspected to have this effect; however, its treatment mechanism for AD requires further study.

There was no significant difference between needle-embedding and acupuncture groups in any of the main evaluation indices (p>0.05); however, it is clinically meaningful that needle-embedding therapy had similar treatment effects as acupuncture. A total of 4 needle-embedding treatments had similar effects to 12 acupuncture treatments, which suggests that needle-embedding treatment may be more clinically convenient than acupuncture, with longer effects and fewer treatments.

These results may be because needle-embedding acupuncture therapy uses a specially designed device to embed certain substances in the body [29], thereby providing a sustained stimulation effect on the Quchi acupoint (LI11).

Additionally, the study participants received a total of 84 acupuncture treatments or 28 needle-embedding treatments. No severe adverse events occurred during the study period. This result shows that both needle-embedding and acupuncture treatments are safe. However, there remains a lack of research regarding needle-embedding treatment; thus, additional research is needed to determine the most appropriate treatment method, number of treatments, interval between treatments, and optimal acupoints.

Further, this study was conducted on a small number of participants, due to limitations of preliminary studies for future clinical trials. Importantly, because participants were randomly assigned, the gender ratio of 1 male and 6 females is not representative of the overall AD population. We plan to determine the number of samples suitable for the purpose of the larger study and to conduct a more complementary and designed clinical trial. Participants will be randomly assigned to 1 of 4 groups (acupuncture, sham acupuncture, needle-embedding, and waiting list) with a 1:1:1:1 allocation ratio and will have scheduled visits during the 8-week treatment period receiving treatment at Quchi acupoint (LI11). Outcome measures will include the Eczema Area and Severity Index (EASI) and blood tests. The outcomes will be evaluated at baseline, as well as at 4, 8, and 12 weeks after subject allocation.

## 5. Conclusions

The SCORAD index, TEWL, skin hydration, and DLQI at 1 week after treatment were significantly improved in both groups (p<0.05). However, there were no significant differences between acupuncture and needle-embedding treatment groups (p>0.05). This study indicates that needle-embedding is not more effective than acupuncture for the treatment of AD, with respect to symptomatic improvement. To the best of our knowledge, this study is the first comparison of the efficacy and safety of needle-embedding treatment with acupuncture treatment for adult AD.

## Figures and Tables

**Table 1:** General characteristics of the participants.

Characteristics	Acupuncture Group (N=7)		Needle-Embedding Group (N=7)		p-value^a^
Gender	N	%	N	%	1.000
Male	1	14.3	1	14.3	
Female	6	85.7	6	85.7	

Smoking	N	%	N	%	0.299
Yes	0	0	1	14.3	
No	7	100	6	85.7	

Drinking	N	%	N	%	0.593
Yes	4	57.1	3	42.9	
No	3	42.9	4	57.1	

Age (Mean ± S.D.)	34.00 ± 5.68		30.71 ± 7.57		0.332

Height (Mean ± S.D.)	162.20 ± 6.26		160.00 ± 5.90		0.688

Weight (Mean ± S.D.)	59.61 ± 5.04		53.44 ± 7.12		0.207

Value are expressed as mean ± SD.

a: comparison between groups: p value from Mann-Whitney *U* test.

*∗*p<0.05.

**Table 2:** Mean separation of SCORAD index.

Evaluation Variable		Acupuncture Group	Needle-Embedding Group	p-value^a^
		Mean ± Standard Deviation		
SCORAD index (n=7 vs. 7)	Baseline	23.9 ± 3.89	26.1 ± 5.24	0.405
	5 Weeks	15.1 ± 3.18	17.0 ± 4.16	
	Difference^b^	8.7 ± 3.06	9.1 ± 5.54	0.697
	p-value^c^	*0.017*	*0.018*	

a: comparison between groups: p value from Mann-Whitney *U* test.

b: 5 weeks after baseline.

c: comparison within groups: p value from Wilcoxon signed rank test.

**Table 3:** Mean separation of Transepidermal water loss (TEWL).

Evaluation Variable		Acupuncture Group	Needle-Embedding Group	p-value^a^
		Mean ± Standard Deviation		
TEWL (n=7 vs. 7)	Baseline	11.7 ± 4.96	9.1 ± 1.52	0.157
	5 Weeks	5.9 ± 1.22	5.4 ± 1.04	
	Difference^b^	5.8 ± 4.25	3.7 ± 1.47	0.306
	p-value^c^	*0.018*	*0.018*	

a: comparison between groups: p value from Mann-Whitney *U* test.

b: 5 weeks after baseline.

c: comparison within groups: p value from Wilcoxon signed rank test.

**Table 4:** Mean separation of skin hydration.

Evaluation Variable		Acupuncture Group	Needle-Embedding Group	p-value^a^
		Mean ± Standard Deviation		
Skin hydration (n=7 vs. 7)	Baseline	24.5 ± 2.40	24.7 ± 3.84	0.749
	5 Weeks	33.7 ± 7.06	34.4 ± 6.40	
	Difference^b^	9.2 ± 6.56	9.7 ± 4.88	0.949
	p-value^c^	*0.018*	*0.018*	

a: comparison between groups: p value from Mann-Whitney *U* test.

b: 5 weeks after baseline.

c: comparison within groups: p value from Wilcoxon signed rank test.

**Table 5:** Mean separation of dermatology life quality index (DLQI).

Evaluation Variable		Acupuncture Group	Needle-Embedding Group	p-value^a^
		Mean ± Standard Deviation		
DLQI (n=7 vs 7)	Baseline	5.3 ± 3.40	5.6 ± 1.90	0.476
	5 Weeks	1.1 ± 0.69	1.0 ± 1.73	
	Difference^b^	4.1 ± 3.40	4.6 ± 2.38	1.000
	p-value^c^	*0.018*	*0.018*	

a: comparison between groups: p value from Mann-Whitney *U* test.

b: 5 weeks after baseline.

c: comparison within groups: p value from Wilcoxon signed rank test.

## Data Availability

The data used to support the findings of this study are available from the corresponding author upon request.
